# Supplementary material for: The interactome of intact mitochondria by cross-linking mass spectrometry provides evidence for coexisting respiratory supercomplexes
Source: Mol Cell Proteomics. 2017 Dec 8;17(2):216–32. doi: 10.1074/mcp.RA117.000470 (PMC5795388; doi:10.1074/mcp.RA117.000470)
Supplement: Supplemental Data [file supp_RA117.000470_133922_0_supp_23978_fzffwf.zip › spectra_annotation/mito_spectra_annotation/142-1-3-1-14-1.pdf]

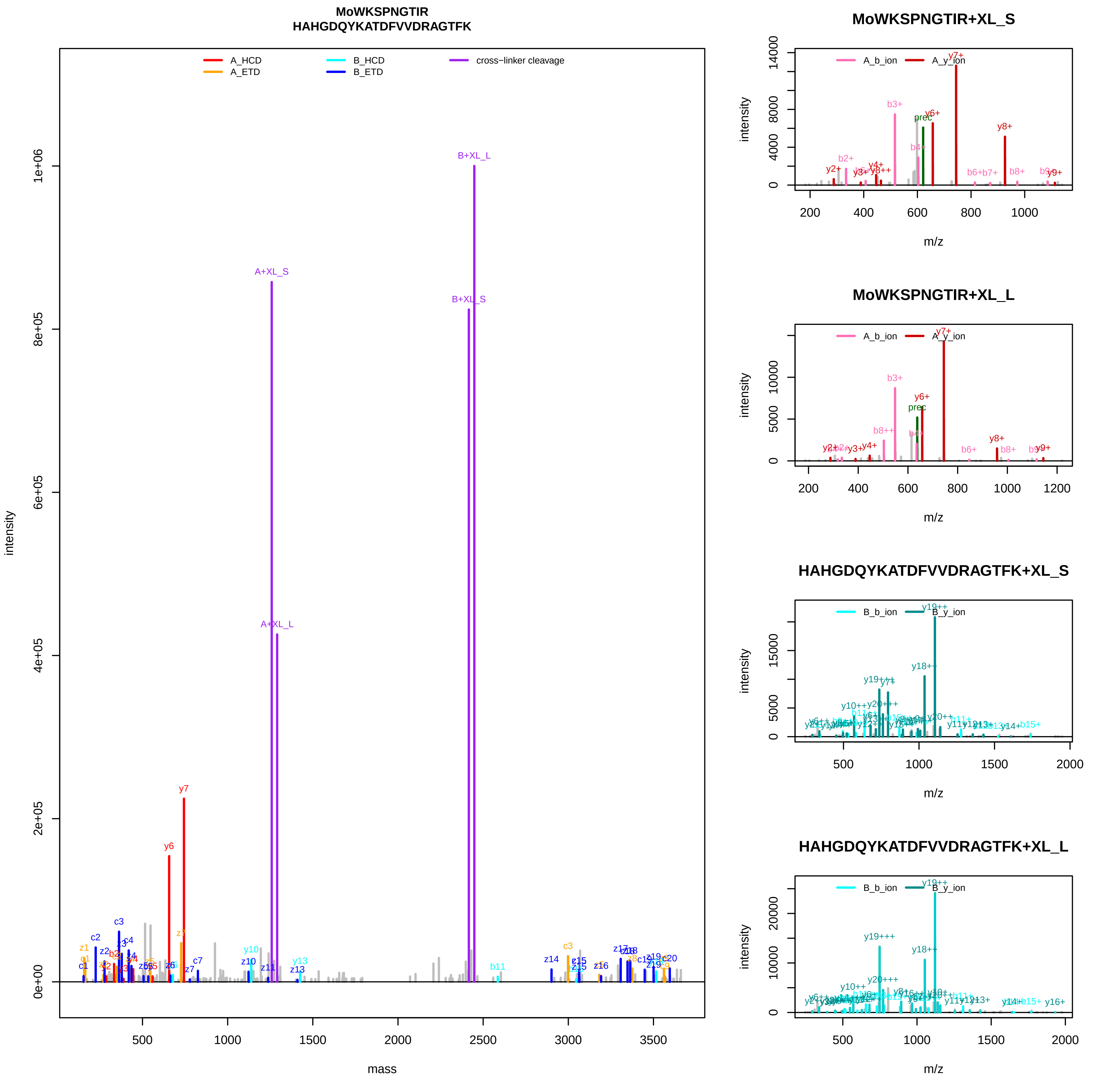

MoWKSPNGTIR
HAHGDQYKATDFVVDRAGTFK
A_HCD
A_ETD
B_HCD
B_ETD
cross−linker cleavage
B+XL_L
A+XL_S
B+XL_S
A+XL_L
y7
y6
z7
c3
c2
z3
c4
z2
z4
c1
c7
z10
y10
z11
y13
z13
b11
z14
z15
c15
z16
z17
z18
c19
z19
c20
intensity
mass
0e+00
2e+05
4e+05
6e+05
8e+05
1e+06
500
1000
1500
2000
2500
3000
3500
MoWKSPNGTIR+XL_S
A_b_ion
A_y_ion
y7+
b3+
y6+
prec
y8+
b4+
b2+
y2+
y4+
y3+
y8++
b6+
b7+
b8+
y9+
MoWKSPNGTIR+XL_L
b8++
HAHGDQYKATDFVVDRAGTFK+XL_S
B_b_ion
B_y_ion
y19++
y18++
y19+++
y10++
y20+++
y20++
y11+
y13+
y14+
b15+
HAHGDQYKATDFVVDRAGTFK+XL_L
y16+
intensity
m/z
